# A Novel, Homozygous c.1502T>G (p.Val501Gly) Mutation in the *Thyroid peroxidase* Gene in Malaysian Sisters with Congenital Hypothyroidism and Multinodular Goiter

**DOI:** 10.1155/2013/987186

**Published:** 2013-04-29

**Authors:** Ching Chin Lee, Fatimah Harun, Muhammad Yazid Jalaludin, Choon Han Heh, Rozana Othman, Sarni Mat Junit

**Affiliations:** ^1^Department of Molecular Medicine, Faculty of Medicine, University of Malaya, 50603 Kuala Lumpur, Malaysia; ^2^Department of Paediatrics, Faculty of Medicine, University of Malaya, 50603 Kuala Lumpur, Malaysia; ^3^Department of Pharmacy, Faculty of Medicine, University of Malaya, 50603 Kuala Lumpur, Malaysia

## Abstract

Congenital hypothyroidism (CH) with multinodular goiter (MNG) is uncommonly seen in children. However, CH associated with goiter is often caused by defective *Thyroid peroxidase* (*TPO*) gene. In this study, we screened for mutation(s) in the *TPO* gene in two siblings with CH and MNG and their healthy family members. The two sisters, born to consanguineous parents, were diagnosed with CH during infancy and received treatment since then. They developed MNG during childhood despite adequate L-thyroxine replacement and negative thyroid antibody screening. PCR-amplification of all exons using flanking primers followed by DNA sequencing revealed that the two sisters were homozygous for a novel c.1502T>G mutation. The mutation is predicted to substitute valine for glycine at a highly conserved amino acid residue 501 (p.Val501Gly). Other healthy family members were either heterozygotes or mutation-free. The mutation was not detected in 50 healthy unrelated individuals. *In silico* analyses using PolyPhen-2 and SIFT predicted that the p.Val501Gly mutation is functionally “damaging.” Tertiary modeling showed structural alterations in the active site of the mutant TPO. In conclusion, a novel mutation, p.Val501Gly, in the *TPO* gene was detected expanding the mutation spectrum of *TPO* associated with CH and MNG.

## 1. Introduction

Congenital hypothyroidism (CH) occurs in babies who are born without the ability to produce adequate amount of thyroid hormones. CH is reported to affect 1 in 3000–4000 life birth [1–3]. Most cases of CH (80–90%) result from dysembryogenesis of the thyroid gland. The remaining 10–20% of cases are due to thyroid dyshormonogenesis or defects in the intermediary steps of thyroid hormone biosynthesis [4]. Studies have shown that thyroid dyshormonogenesis is associated with defects in genes that are involved in thyroid hormone synthesis. Mutations in the *TPO* are one of the most common causes of dyshormonogenetic CH disorder [5]. Other candidate genes include dual oxidase 2 (*DUOX2*) [6], solute carrier family 5 or sodium iodide symporter (*SLC5A/NIS*) [7], thyroglobulin (*TG*) [8], and thyroid stimulating hormone receptor (*TSHR*) [9].

The *TPO* gene is located on chromosome 2p25 spanning at least 150 kb and contains 17 exons [10]. The full-length of the mRNA transcript, TPO1, is 3152 bp (GenBank accession number NM_000547.5) which encodes 933 amino acids. TPO is tissue-specific [11] and the mature TPO protein is organized into a large extracellular fragment, a short transmembrane segment and a short cytoplasmic tail [11]. TPO is a heme-containing protein that catalyzes the iodination of thyroglobulin and the coupling of some of the iodotyrosyl residues to generate active iodothyronines, T_3_ and T_4_ [12, 13]. To date, more than 60 mutations in the *TPO* gene have been described. The majority of the mutations were localized in exons 8, 9, and 10 of the gene that encode the catalytic heme binding domain of the protein [14, 15].

Cases of CH due to defects in the *TPO* gene were usually associated with a recessive inheritance pattern [16]. However, an autosomal dominant inheritance pattern had also been reported [17]. In the present study, we characterized mutation(s) in the *TPO* gene in two siblings with dyshormonogenetic CH and MNG. The effects of the mutation on protein function were predicted using *in silico* analyses as TPO protein synthesis is tissue-specific and thyroid tissues from both sisters were not available. 

## 2. Subjects and Methods

### 2.1. Subjects and Family Members

Two sisters of Malaysian-Indian origin were diagnosed with CH during infancy, with both having no cord TSH screening performed at birth (not introduced as a national policy at that time). The proband, 11-1, was diagnosed with CH at 4 months old when she presented with classical signs of hypothyroidism. At presentation, serum TSH was more than 160 *μ*IU/mL (Normal range: 0.3–5.0 uIU/mL) and total T_4_ less than 13.0 nmol/L (Normal range: 64–167 nmol/L), respectively. Her younger sister, II-3, was born at a private maternity centre in 1997 at 36 weeks of gestation with no cord TSH screening as well. She was detected to have CH at 2 weeks old when she was screened during her hospital stay for birth asphyxia, with the background history of elder sister with CH. At diagnosis, her free T_4_ was 2.1 pmol/L (normal range: 11.5–23.2 pmol/L). They both received thyroid replacement with L-thyroxine. Technetium-99m thyroid scintigraphy was performed when they were 3 years old when L-thyroxine was temporarily stopped for 4 weeks and thyroid function repeated. The thyroid scan revealed the presence of glands. However, the TSH rose markedly in both patients with decrease in free T_4_ confirming the need for life-long replacement.

 In the course of their follow-up clinic visits, both patients 11-1 and 11-3 developed multinodular goiters at 11 years and 10 years, respectively. This was confirmed by thyroid ultrasonography. Both had negative thyroid antibody screening. However, the human thyroglobulin (hTG) level of 11-1 was 137 ng/mL (normal: 0–55 ng/mL) while the hTG level of II-3 was markedly elevated to 1284 ng/mL. Both had fine-needle aspiration and cytology (FNAC) of their goiter, which revealed no evidence of malignancy. Patient 11-1 suffered as a consequence of the late treatment, affecting her growth and cognitive function with IQ of 50–55 (normal IQ = 85–115). Her younger sister, 11-3, had normal physical growth and an IQ of 80 which is slightly below the average IQ range of 85–115. No other congenital anomalies were detected. Their parents (I-1 and I-2) who are consanguineous (father is the maternal uncle) and the other two proband's siblings (II-2 and II-4) were all healthy, euthyroid with the absence of congenital anomalies. Written informed consent was obtained from the parents for their blood and their children's blood for genetic analysis of the *TPO* gene. This study was approved by the University of Malaya Medical Centre (UMMC) Ethical Committee (Institutional Review Board) in accordance to the ICH-GCP guideline and the Declaration of Helsinki (reference number, 654.16).

### 2.2. Mutational Analysis of the *TPO* Gene

Peripheral venous blood samples were collected from the proband, the affected sister, and their healthy family members. Genomic DNA was extracted from peripheral blood leukocytes using QIAamp DNA Blood Mini Kit (Qiagen, Germany) according to the manufacturer's protocol. The *TPO *gene was PCR-amplified with flanking intronic primers [18, 19] covering all the exons including the hotspot mutation regions, exons 8, 9, 10, 12 and 14. A 50 *μ*L of PCR reaction mixture containing 100–250 ng of genomic DNA, 1X *Taq* Buffer with KCl, 2.5 mM of MgCl, 200 *μ*M of dNTP, 8% dimethyl sulfoxide (DMSO), 1 unit of *Taq* DNA polymerase (Fermentas, USA), and 20 pmol of each forward and reverse primers were prepared. Thirty five cycles of amplification were carried out with standard PCR protocol at annealing temperature of 55°C for all coding exons for 30 seconds. PCR products were purified using QIAquick PCR purification kit (Qiagen, Germany) following the manufacturer's instructions. The purified PCR products were sequenced using ABI Prism Gene Sequencer, Model 3100, Version 3.7 (Research Biolabs, Singapore). To confirm that an alteration in the *TPO* gene is not a polymorphism, a total of 100 chromosomes from 50 unrelated healthy individuals were also screened for the same mutation. Thyroid tissue samples from the patients were not available; therefore, *in silico* analyses were carried out to predict the impact of the mutation on protein structure and function.

### 2.3. *In Silico* Analyses

Protein sequence alignment across species was performed using CLC Sequence Viewer 6.5.2 software (CLC bio, Aarhus, Denmark). The potential functional impact of p.Val501Gly was predicted using Polymorphism Phenotyping 2 (PolyPhen-2) [20], at http://genetics.bwh.harvard.edu/pph/ and Sorting Intolerant from Tolerant (SIFT) [21], at http://sift.jcvi.org/www/SIFT_enst_submit.html. Both PolyPhen-2 and SIFT are online prediction tools which predict the degree of (possible) impact of an amino acid substitution on the structure and function of a human protein. This functional impact of the mutation was predicted as “benign,” “possibly damaging,” “probably damaging,” or “unknown” for PolyPhen and as “tolerated” or “damaging” for SIFT.

The crystal structure of human TPO is currently not available; hence, in this study, its homology model was generated. Using the online protein-protein BLAST program [22, 23] from National Center for Biotechnology Information (NCBI) website (http://blast.ncbi.nlm.nih.gov/Blast.cgi), the human TPO protein was found to have the highest amino acid homology to that of sheep lactoperoxidase, where fortunately, the crystal structure of the latter is available (PDB id: 2IKC). Thus, a homology model was generated with 2IKC as a template using Modeller 9.10 software [24]. Using the same software, a homology model of the human TPO containing the p.Val501Gly mutation was also generated for structural comparison. Both models were then subjected to molecular dynamics simulation using Amber 10 software [25, 26] with FF99SB force field [27], explicit water environment at 310 Kelvin and run for 10 ns. Structures with the lowest potential energy were extracted, verified using Ramachandran plots which were generated by Procheck software (http://nihserver.mbi.ucla.edu/SAVES) to check the stereochemical quality of the protein [28], Verify3D software to determine the compatibility of the 3D atomic model with its own 1D amino acid sequence [29], and Errat software (http://nihserver.mbi.ucla.edu/SAVES) to analyse the statistics of nonbonded interactions between different atom types [30].

## 3. Results

### 3.1. Mutational Analysis of the *TPO* Gene

**Figure 1:**
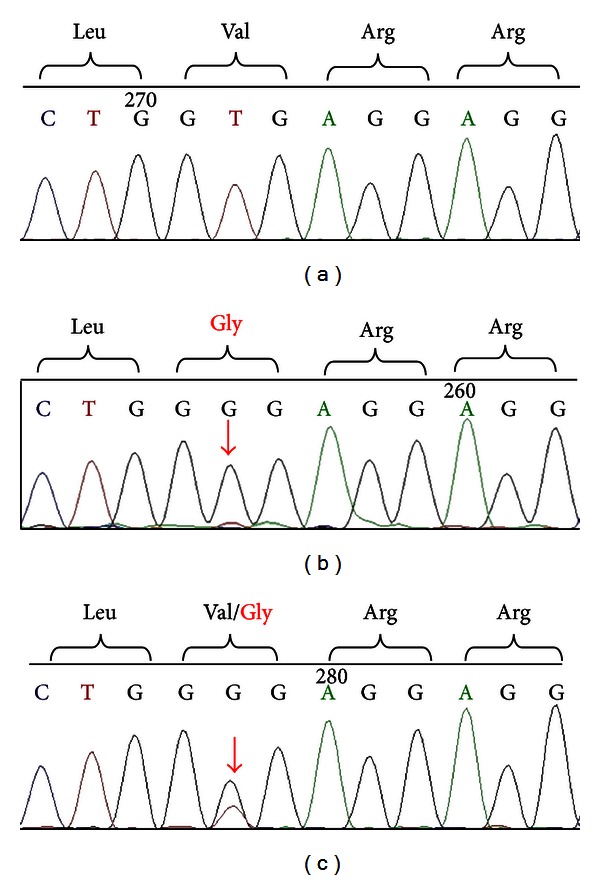
DNA sequencing profiles. Electropherogram profiles of II-4 with a wild type allele (a), II-1 who was a homozygous for the c.1502T>G (b), and I-1 who was a heterozygous for the c.1502T>G (c). The single nucleotide transition at location 1502 bp (c.1502T>G) is indicated by an arrow. The nucleotide change is predicted to cause an amino acid substitution of valine by glycine at residue 501 (p.Val501Gly).

**Figure 2:**
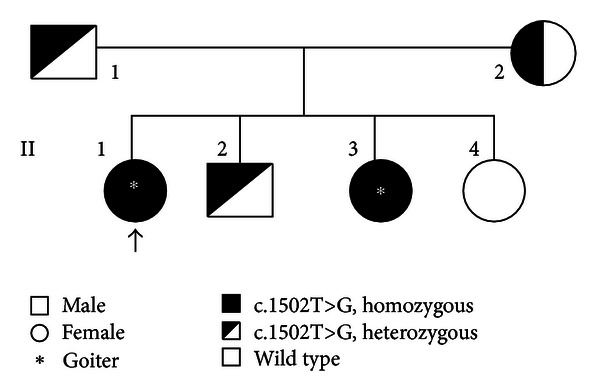
Family pedigree of the index patient. A family pedigree of two sisters with congenital hypothyroidism and multinodular goiter. The proband is indicated by an arrow. N/A: data are not available; TSH: thyrotropin; TT_4_: total thyroxine; FT_4_: free thyroxine; Tg, thyroglobulin.

**Figure 3:**
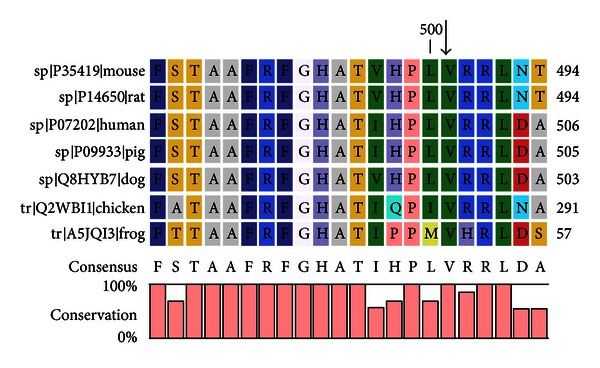
Multiple-sequence alignment of human TPO with those of mouse, rat, pig, dog, chicken, and frog. The alignment data show that valine at position 501 is highly conserved amongst human and many different animal species. The position of the mutated residue (p.Val501Gly) is indicated by the arrow. This figure was generated using the CLC Sequence Viewer 6.5.2 algorithm.

DNA sequencing of exons 1 to 17 of the *TPO* gene in the index patient, II-1, revealed a novel homozygous mutation, c.1502T>G (Figure 1). The mutation is predicted to cause a change from valine to glycine at amino acid 501 (p.Val501Gly). In addition, other documented polymorphisms (12A>G, c.769G>T, c.1193G>C, c.2145C>T, c.2173A>C, and c.2540T>C) in exons 1, 7, 8, 12, and 15, respectively, were also detected in II-1. The c.1502T>G mutation was not found in 100 chromosomes from 50 unrelated healthy individuals. Further DNA sequencing analysis revealed that her affected sibling, II-3, was also homozygous for the same mutation. On the other hand, both parents and one of the index patient's siblings (II-2) were found to be heterozygous for the mutation. No mutation was detected in another sibling, II-4. A family tree of the patient showing the mode of inheritance of the mutation is shown in Figure 2. Amino acid alignment of the human TPO proteins with mouse, rat, pig, dog, chicken, and frog indicated that Val-501 residue is located within a highly conserved region (Figure 3).

### 3.2. *In Silico* Predictions of Functional Impact of the c.1502T>G on the *TPO *Protein

**Figure 4:**
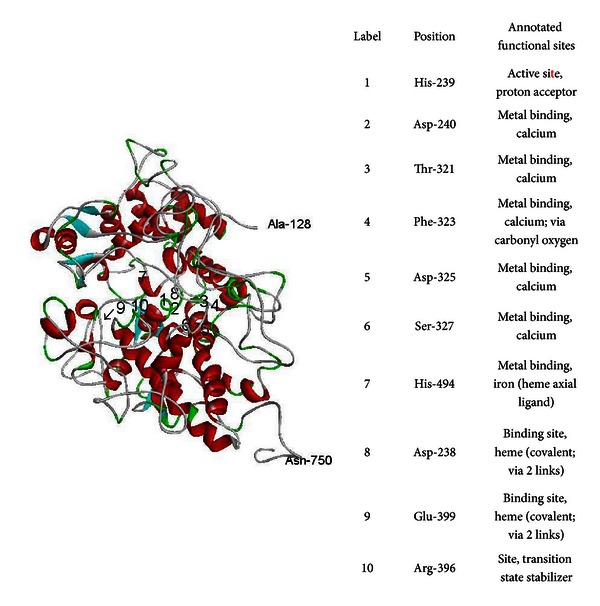
Homology model of the wild type human TPO using sheep lactoperoxidase as a template (PDB_2IKC). The positions of the annotated functional site referred from UniProt database (reference number: P07202). The arrow indicates the positions of the mutated residue (p.Val501Gly).

*In silico* analyses using PolyPhen-2 and SIFT predicted that the p.Val501Gly mutation is functionally “possibly damaging” (HumVar score of 0.827) and “damaging” (SIFT score of 0), respectively (data not shown). The generated three-dimensional homology models based on 21KC template (47% sequence similarity) covered 62% of the full sequence of TPO protein (Figure 4). Ramachandran plots from Procheck showed that the wild type and the mutant homology models had 2 and 7 amino acid residues located in the disallowed regions (regions which are disallowed for all amino acids due to steric clashes, except for glycine which lacks a side chain), respectively. However, these amino acid residues were distant from the active site. Verify3D showed that the wild type and mutant models had passed as being good models by having 87.2% and 90.8% of residues with an average 3D to 1D score of more than 0.2, respectively. Errat's results indicated that the two models had overall quality factor of about 88 over 100 (the decrease in value is due to flexible loop regions which could not be aligned with available crystal structures). Consequently, the two homology models were acceptable for subsequent analyses.

**Figure 5:**
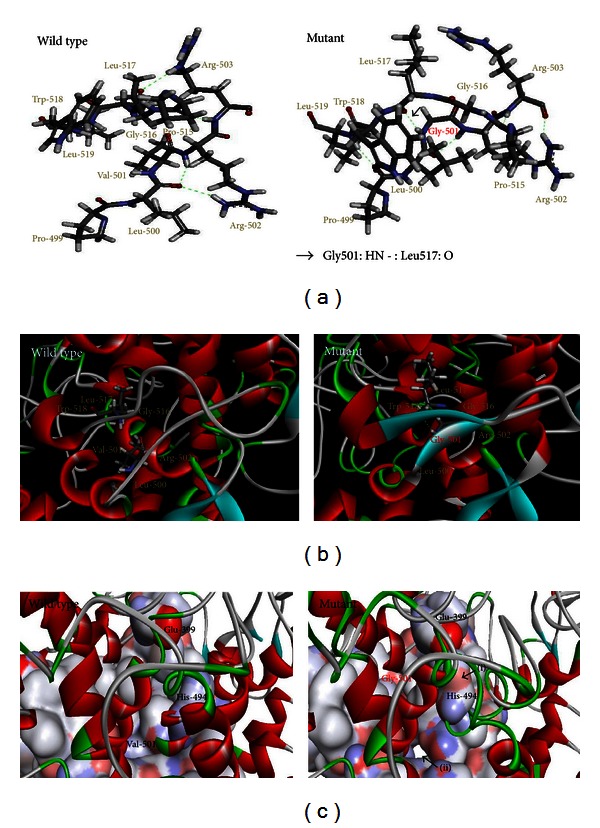
Computer generated models of the wild type and mutant TPO. (a) Residues Pro-515 to Leu-519 and Pro-499 to Arg-503 are displayed in sticks for wild type and p.Val501Gly mutant TPO proteins. H-bonds are shown as green dashed lines. For the mutant protein, H-bond Gly501: HN -:Leu517: O is indicated by an arrow. (b) Ribbon presentation of wild type and p.Val501Gly mutant TPO proteins. The residues Val-501, Gly-501, and Leu-517 are shown as sticks. The H-bond between the backbone oxygen of Leu-517 and amide hydrogen of Gly-501 in the mutant contributed to the formation of a beta sheet that comprised two beta strands from Leu-500 to Arg-502 and Gly-516 to Trp-518. (Red areas represent alpha helix, cyan shows the beta-pleated sheet areas, gray areas represent coils in the protein, and green areas represent the turn.) (c) Connoly surface representation of the internal surface of TPO proteins and the binding site of heme (Glu-399) and iron (His-494) in wild type and p.Val501Gly mutant. Residues on the top layer are shown as ribbon to allow the visualization of the binding pocket. Significant structural alterations are indicated by arrows: (i) conformational changes in the heme/iron binding pocket and (ii) small “opening” from external surface. The Connolly surface is coloured according to electrostatic potential spectrum.

Comparison between predicted tertiary structures of the wild type and mutant proteins revealed that the H-bond between the backbone oxygen of Leu-517 and amide hydrogen of mutant residue Gly-501 (Figure 5(a)) contributed to the formation of a beta sheet in the mutated region (Figure 5(b)), which requires beta strands to be connected laterally by hydrogen bonds. Significant structural alterations in the annotated binding pocket of heme (Glu-399) [31] and iron binding site (His-494) [32] were also detected in the p.Val501Gly mutant (Figure 5(c)).

## 4. Discussion

MNG is not common in children [33]. MNG associated with CH, which is more uncommon, is often caused by defective *TPO* gene [15, 34, 35]. In the present study, we identified a novel, homozygous c.1502T>G mutation in the *TPO* gene in two siblings from consanguineous parents, with severe goitrous CH. Family members with heterozygous p.Val501Gly were asymptomatic supporting the recessive inheritance pattern [16]. The mutation was not detected in 100 chromosomes from 50 normal unrelated individuals suggesting that the c.1502T>G alteration is not a polymorphism.

Val-501 is conserved amongst many animals including mice and rats implying its importance in the structure/function of the TPO. Valine is seldom directly involved in protein function due to its very nonreactive side chain [36]. Alteration of amino acid in this region is more likely to affect the protein conformation. Moreover, replacing valine with glycine might lead to less hydrophobic interactions as the former has a larger aliphatic side chain while the latter has H as its side chain. Nonetheless, tertiary modeling showed structural alterations in the TPO mutant, suggesting the mutation could cause structural instability of the protein.

A proposed iron (heme axial ligand) binding site, His-494 [32], is located six residues away from the mutation site. The early step in the biosynthesis of thyroid hormones involves the oxidation of the iodide (I^−^) to iodinium (I^+^) ions by TPO in the presence of H_2_O_2_ [37]. The heme iron in the TPO plays an important role in the redox reaction. In this study, 3D analysis showed that the iron binding site of the p.Val501Gly mutant TPO is slightly exposed than that of the wild type (Figure 5(c)). It is very likely that the additional entry in the proposed binding pocket of mutant TPO would interfere with the binding of the gas ligand and/or cause improper conformational changing after binding. Alternatively, the absence of a relatively bulky aliphatic side chain, due to Gly-501 lacking a side chain in p.Val501Gly mutant TPO, could alter the hydrophobic pocket and thus could interfere with the heme binding at Glu-399. Interestingly, Rivolta et al. in 2003 reported that p.Pro499Leu mutation in the TPO which is also located within the hydrophobic pocket was associated with severe goitrous CH [18]. We therefore believed that this region is significantly important for the normal activity of the protein. The mechanism by which p.Val501Gly in the TPO may induce MNG remains to be elucidated, although the tertiary structure of the protein appeared to be modified. Considering that there is an increased risk of thyroid carcinoma in MNG patients [38], it is important to have a careful surveillance for potential thyroid neoplasm in these patients.

## 5. Conclusion

In conclusion, we demonstrated a novel, homozygous c.1502T>G mutation (p.Val501Gly) in the *TPO* gene which could be associated with dyshormonogenetic CH and MNG in members of a Malaysian-Indian family.
